# Development of facial palsy following COVID-19 vaccination: A systematic review

**DOI:** 10.1016/j.amsu.2022.104758

**Published:** 2022-09-30

**Authors:** Maman Khurshid, Iflah Ansari, Hafsa Ahmad, Hafsa Ghaffar, Aiman Khurshid, Abia Shahid, Mohammad Yasir Essar, Irfan Ullah, Huzaifa Ahmad Cheema

**Affiliations:** a Department of Internal Medicine, Dow University of Health Sciences, Karachi, Pakistan; b Department of Internal Medicine, Baqai Medical University, Karachi, Pakistan; c Department of Forensic Medicine, Civil Hospital, Karachi, Pakistan; d Department of Medicine, King Edward Medical University, Lahore, Pakistan; e Kabul University of Medical Sciences, Kabul, Afghanistan; f Department of Internal Medicine, Kabir Medical College, Gandhara University, Peshawar, Pakistan

**Keywords:** COVID-19 vaccination, Oxford-AstraZeneca, Pfizer Pfizer–BioNTech, Facial palsy, Bell's palsy

## Abstract

**Objective:**

Reports of facial palsy occurring after the receipt of COVID-19 vaccines have raised concerns but are rare. The purpose of this study is to systematically assess the association between COVID-19 vaccination and facial palsy.

**Methods:**

Our systematic review followed the Preferred Reporting Items for Systematic Reviews and Meta-analyses (PRISMA) checklist and compiled all the reported cases of facial palsy post-COVID-19 vaccination. We discussed the probable pathophysiology behind facial palsy as a consequence of COVID-19 vaccination and measures to be taken for future reference. Furthermore, we conducted a detailed assessment of characteristics, clinical courses, treatment, and recovery of patients with facial palsy after receiving a COVID-19 vaccine.

**Results:**

We included 37 studies providing data on 58 individuals in our review. Over half (51.72%) of the patients complained of facial paralysis following the Oxford-AstraZeneca vaccination. Out of 51 cases, most (88.24%) occurred after the 1st dose. The majority (53.45%) of cases had bilateral facial palsy. Intravenous immunoglobin (IVIg), corticosteroids, and plasmapheresis were the first line of treatment with 75.93% of patients partially recovered, including those undergoing treatment or a lack of follow-up till the end while 22.22% had complete symptomatic recovery.

**Conclusions:**

Our review shows that Bell's palsy can be a plausible non-serious adverse effect of COVID-19 vaccination. However, the association observed between COVID-19 vaccination and Bell's palsy is less threatening than the COVID-19 infection. Hence, vaccination should be encouraged because facial palsy, if it occurs, has shown favourable outcomes with treatment.

## Highlights

• Facial palsy has been reported as a rare adverse event of COVID-19 vaccination.
• Over half of the cases occurred following the Oxford-AstraZeneca vaccination.
• In majority of the patients, the onset of symptoms was after the 1st dose of the vaccine.
• The presentation is mild, and most of the patients recover either completely or partially.
• Autoimmunity (molecular mimicry) is hypothesized to be the major mediator in pathogenesis.

## 1 Introduction

Bell's palsy, commonly known as idiopathic facial paralysis (IFP) is a non-progressive neurological disorder occurring in almost 40,000 individuals each year in the United States of America (USA) [1]. The condition involves inflammation of the seventh cranial nerve (facial nerve) supplying the muscles of the face and parasympathetic innervation of lacrimal and salivary glands and limited sensory fibres to the anterior two-thirds of the tongue. Hence, patients with this disorder usually present with facial stiffness, inability to control facial expressions, failure to close the eye (lagophthalmos), drooling, tearing up, and loss of sense of taste in the anterior two-thirds of the tongue (ageusia). The disease has no predilection for gender or either side of the face, however, rarely bilateral Bell's palsy (facial palsy) is also noted [2]. It is believed that inflammation at the level of the geniculate ganglion is the culprit pathology. This increases pressure in the fallopian canal leading to compression, ischemia, and possibly demyelination of the nerve. In most cases, facial paralysis is temporary and resolves after treatment with steroids and antivirals but up to 30% of patients can develop long-term complications while 5% can have a high degree of sequelae like permanent facial paralysis or synkinesis [3]. Classically, the cause of facial nerve inflammation is still unknown, however, some association has been described with Herpes Simplex Virus Type I (HSV-1), Lyme Disease, Guillain-Barre Syndrome, autoimmune conditions like sarcoidosis, and influenza vaccine. Recently, various cases of facial palsy after the COVID-19 vaccination have also been reported [[4], [5], [6], [7]].

COVID-19 is an acute respiratory illness caused by SARS-CoV-2. Since its intimation, this deadly pandemic has infected approximately 505,035,185 individuals and caused 6,210,719 deaths worldwide [8]. To curb the rapid spread of this disease research was initiated on diagnosis, prevention, and treatment modalities for coronavirus. Drugs like hydroxychloroquine, remdesivir, favipiravir, and tocilizumab were explored for their safety and efficacy, however, there was no definitive treatment. The game changed when the Pfizer-BioNTech (BNT162b2) mRNA vaccine was authorized for emergency use by the US Food and Drug Administration (FDA) in December 2020 [9]. Currently, approved vaccines for COVID-19 include Comirnaty (BNT162b2), Spikevax (mRNA-1273), Oxford-AstraZeneca COVID-19 Vaccine (AZD1222), Sputnik V, Johnson & Johnson/Janssen COVID-19 Vaccine (JNJ-78436735; Ad26.COV2. S), CoronaVac, Covaxin (BBV152) and 23 others [10]. Due to the emergency, vaccines were granted approval based on only the initial phases of clinical trials without completion of all the phases of a clinical trial [11,12]. Thus, it is important to monitor adverse events reported post-COVID-19 vaccination. The Pfizer-BioNTech and Moderna vaccine trials revealed seven cases of Bell's palsy in comparison with just one in the control groups [13,14]. The 7 (P = 0.07) rate ratio suggests a possible link between the COVID-19 vaccination and Bell's palsy. Additionally, Ozonoff et al. reported that the incidence of Bell's palsy in the mRNA vaccines was 3.5–7 times higher than in the general population [15]. A Hong Kong study reported an increased overall risk of Bell's palsy after CoronaVac, an inactivated vaccine [16]. Dutta et al. also reported 19,529 neurological adverse events after COVID-19 vaccination, including facial paralysis [17].

On the other hand, a recent disproportionality analysis of the World Health Organization (WHO) pharmacovigilance database by Renoud et al. indicated that the rate of facial paralysis reported after mRNA COVID-19 vaccination is not higher than the observed rate with influenza and other viral vaccines [18]. However, this does not completely rule out a possible association and that study does not have a risk estimation as the population exposed to the vaccine is unknown. Similarly, a hospital-based study suggested no connection between the Pfizer BNT162b2 vaccine and Bell's palsy [19].

In light of the existing data, it is clear that the association between Bell's palsy and COVID-19 vaccination is disputed while the literature is sparse. Furthermore, available evidence has not been studied or summarised to portray a potential relationship between the two. It is imperative to review the present resources on this neurological disorder to better understand and prevent its future incidence. Moreover, data from the U.S. Census Bureau's Household Pulse Survey (HPS) on hesitancy rates for COVID-19 vaccination showed hesitancy rates ranging from 2.69% to 26.7% across the US [20]. To reduce reluctance and ensure maximum vaccination of COVID-19, we need to thoroughly study reported adverse events, their severity, and how to tackle them. In this study, we systematically reviewed all reported cases of facial palsy after the COVID-19 vaccination. We also discussed the plausible pathophysiology behind facial palsy as a result of COVID-19 vaccination and what this implies for the future. Furthermore, we conducted a detailed analysis of characteristics, clinical courses, treatment, and recovery of patients affected with facial palsy after vaccination for COVID-19.

## 2 Methods

### 2.1 Literature search

Our study followed the Preferred Reporting Items for Systematic Reviews and Meta-analyses (PRISMA) checklist (Supplementary File 1) [21]. We searched for relevant articles on PubMed and Google Scholar databases from the onset of the COVID-19 pandemic till April 22, 2022. Boolean operators and keywords/subject headings synonymous with facial palsy (e.g., facial paralysis OR facial paresis OR Bell's palsy OR facial weakness) AND COVID-19 vaccine (e.g., COVID-19 Vaccines OR SARS-CoV-2 Vaccines OR Coronavirus Disease 2019 Vaccines OR 2019-nCoV Vaccine OR SARS Coronavirus 2 Vaccines) were used for literature search on respective databases. The references of selected studies were also verified to ensure the completeness of the search. The complete search strategy is given in Supplementary File 3. Furthermore, our study was registered in the International Prospective Register of Systematic Reviews (PROSPERO) bearing registration number: CRD42022328860 [22].

### 2.2 Inclusion and exclusion criteria

We included all the studies which recorded patient-level data of individuals who developed facial palsy post- COVID-19 vaccination. Reviews, meta-analyses, and literature with aggregate-level data were excluded. Moreover, studies were excluded if they had insufficient data on the clinical progression of the condition. Additionally, there was no language restriction, and all published literature was reviewed irrespective of its language. Two authors (HA and IA) screened the title, abstract and full texts of studies in duplicate. Any conflict in the study selection was, thereafter, resolved by a senior author (MK).

### 2.3 Data extraction

Selected studies were transferred to an Excel sheet where after, tables were formed after the removal of duplicate studies. Extracted data came under the headings of author name, study type, history/comorbidities, age, gender, Guillain Barre present (yes/no), affected side of the face, COVID-19 vaccine name/type, dose number preceding facial palsy (dose 1 or 2), the onset of facial symptoms following last vaccination (days), clinical features of facial palsy, other complaints, examination results (physical and neurological exams), findings in cerebrospinal fluid (CSF) analysis findings (protein level and cell count), investigations, treatment provided and treatment outcome. Additionally, the sole findings of two imaging techniques, magnetic resonance imaging (MRI) and computed tomography (CT), were chosen for inclusion in our tables. The selected studies varied in quality and no set criteria for assessment or reporting of the condition was devised. Consequently, inconsistencies were noted in their reporting methods.

### 2.4 Quality assessment

Joanna Brigg's Institute Critical Appraisal Checklist for Case Reports and Case Series was used for the quality assessment of included studies [23,24]. For quality assessment, the seven included LTEs were also treated as either case reports or case series based on the number of cases reported. Each qualitative answer was converted into a numeric score. Quality assessment was conducted independently by two reviewers (IA and HG) and the final score was given after resolving disagreements. Different tools were used based on the study type of every included study. Case reports and case series had 8 and 10 questions, respectively. Included case series (n = 7) had a mean score of 8.86 ± 0.64 with scores ranging from 8 to 10 [[25], [26], [27], [28], [29], [30], [31]]. Meanwhile, the 30 case reports had scores ranging from 5 to 8 with a mean score of 6.57 ± 0.76 [[4], [5], [6], [7],[32], [33], [34], [35], [36], [37], [38], [39], [40], [41], [42], [43], [44], [45], [46], [47], [48], [49], [50], [51], [52], [53], [54], [55], [56], [57]]. A detailed quality assessment is provided in Supplementary File 3. Furthermore, we also used the A Measurement Tool to Assess Systematic Reviews (AMSTAR 2) checklist to assess the quality of our systematic review which came out to be “moderate” (Supplementary File 2) [58].

### 2.5 Statistical analysis

Our study provided comprehensive data on individuals who experienced facial paralysis following COVID-19 vaccination. This included information on study type, patient characteristics, both facial palsy and non-facial palsy-related complaints, diagnostic test results, treatment regimen, and outcome of the treatment regimen (Table 1). Means and standard deviations (SD) of age were calculated while other variables were expressed as a percentage of their total number of responses.

**Table 1:** Characteristics of included studies.

S #	Author	Study type	No of cases	Patient S#	Past History/comorbids	Age/sex	GBS Present?	Affected side of face	COVID-19 vaccine name/type	Which dose led to a symptom?	onset of facial symptom after last vaccination	Clinical features of facial palsy	Other complaints	Examination results	CSF analysis results	Investigation	Treatment	Treatment outcome
1	Colella et al.	LTE	1	1	None	37/M	No	Left	Pfizer-BioNTech	1	5 days	left facial droop, lagophthalmos and mild labial hypomobility with flattening of forehead's skin and nasolabial fold.	Malaise, fatigue, headache, left latero-cervical pain and monolateral muscular weakness	N/A	N/A	N/A	prednisone, eye drops and night time eye dressing	PR
2	Finsterer et al.	Case report	1	1	GBS	32/M	Yes	Right	vector-based vaccine	1	8 days	dysphagia	peripheral limb paraesthesia, muscle weakness and headache	Neurologic: Right peripheral facial palsy affecting orbicularis oris muscle, motor: B/L muscle weakness and DTR reduced	Protein: elevated	MRI brain and cervical spine: B/L few nonspecific T2-hyperintensities in the white matter	IVIg and plasmapheresis	PR
3	Repajic et al.	Case report	1	1	Bell's palsy and HTN	57/F	No	Left	Pfizer-BioNTech	2	1 day	left facial droop and lagophthalmos, left ear otalgia and aguesia	jaw pain	Neurologic: consistent with CN7 palsy; motor, sensory, gait and cerebellar examination normal.	N/A	N/A	prednisone and antivirals	CR
4	Nishizawa et al.	Case report	1	1	T2DM, HTN and hyperlipidemia	62/F	No	Right	J&J/Janssen COVID-19 vaccine	N/A	20 days	Right facial paralysis, lagophthalmos HB grade VI	None	Physical: consistent with facial palsy; motor, sensory, gait and cerebellar examination normal.	N/A	Head CT and brain MRI: unremarkable	N/A	N/A
5	Martin-Villares et al.	LTE	1	1	Bell's palsy	34/F	No	Right	Moderna COVID-19 vaccine	1	2 days	right facial pain, facial palsy HB grade III	None	N/A	N/A	MRI: unremarkable	Deflazacort, eye support, facial rehabilitation	CR
6	Maramattom et al.	Case series	7	1	None	43/F	Yes	B/L	Oxford-Astrazeneca	1	after 20 days	facial diplegia	upper back pain, areflexic quadriparesis and respiratory failure	sensory: normal; motor strength: severely weakened muscle power and areflexia	Albuminocytological dissociation	N/A	IVIg and MV	CR
				2	N/A	67/F	Yes	B/L	same	1	16 days	facial diplegia and dysphagia	distal paraesthesia, limb weakness and respiratory failure	CN: Right abducens palsy, facial diplegia, and bulbar palsy Sensory: abnormal; motor strength: severely weakened muscle power and areflexia	Albuminocytological dissociation	MRI Brain: unremarkable	MV, IVIg and plasmapheresis	PR
				3	N/A	53/F	Yes	Right	same	1	12 days	facial and tongue numbness	B/L LL numbness, weakness, back pain and Respiratory failure	CN: Right facial and tongue numbness, facial diplegia. Sensory - B/L LL distal sensory impairment (pinprick and vibration), right trigeminal V2–V3 sensory impairment (touch, pinprick). Areflexia	Albuminocytological dissociation	MRI Brain: unremarkable	MV and IVIg	PR
				4	N/A	68/F	Yes	B/L	same	1	18 days	B/L facial numbness, LMN facial weakness and dysphagia	B/L UL and LL numbness, weakness, areflexic flaccid quadriplegia and respiratory failure	CN: facial diplegia, bulbar palsy, B/L facial numbnessSensory: B/L distal UL and LL numbness, (distal LL pinprick impairment), B/L sensory impairment to touch in all 3 divisions of the trigeminal nerve, areflexia	Albuminocytological dissociation	MRI Brain: unremarkable	MV and IVIg	PR
				5	N/A	70/M	Yes	B/L	same	1	11 days	facial diplegia, B/L facial numbness and bulbar palsy	B/L distal UL and LL numbness and respiratory failure	CN: facial diplegia, bulbar palsySensory: B/L distal UL and LL numbness, no objective sensory impairment and areflexia	N/A	N/A	MV and IVIg	PR
				6	N/A	69/F	Yes	B/L	same	1	12 days	facial diplegia and bulbar palsy	B/L distal UL and LL numbness, complete ophthalmoplegia leading to left Abducens nerve palsy	CN: facial diplegia, bulbar palsy, complete ophthalmoplegia, Sensory: B/L UL and LL distal numbness, no objective sensory impairment and areflexia	N/A	N/A	IVIg and Plasmapheresis	PR
				7	N/A	69/F	Yes	B/L	same	1	13 days	facial diplegia and bulbar palsy	B/L UL and LL numbness and respiratory failure	CN: facial diplegia, bulbar palsySensory: B/L UL and LL numbness, no objective sensory impairment and areflexia	albuminocytological dissociation	N/A	MV and IVIg	PR
7	Allen et al.	Case series	4	1	None	54/M	Yes	B/L	Oxford-Astrazeneca	1	16 days	bifacial weakness	distal dysesthesia in feet and hands	DTR normal, no objective sensorimotor signs. Cerebellar, bulbar, extraocular movement and respiratory function normal. No dysautonomia General exam: unremarkable	cell count: elevated; Protein: elevated	MRI brain: unremarkable	oral Prednisolone	PR
				2	ulcerative colitis	20/M	Yes	B/L	same	1	26 days	Bifacial weakness	occipital headache, dysesthesia in distal LL	neck movement uncomfortable, remainder of the neurological examination normal. DTR normal, no objective sensorimotor signs. Cerebellar, bulbar, extraocular movement and respiratory function normal. No dysautonomia General exam: unremarkable	cell count: elevated; Protein: elevated	MRI Brain: unremarkable	oral Prednisolone	PR
				3	asthma and osteoarthritis with B/L knee replacement	57/M	Yes	B/L	same	1	21 days	bifacial weakness, dysarthria	lumbar back pain that radiated to flanks, distal dysesthesia in feet, proximal leg weakness	subjective diplopia on extreme left gaze, normal extraocular eye movements. Symmetric weakness proximally in legs. DTR absent at the knees but normal elsewhere.	cell count: elevated; Protein: elevated	Noncontrast MRI brain: unremarkable	IVIg	PR
				4	HTN	55/M	Yes	B/L	same	1	29 days	facial diplegia	B/L thigh paresthesias, sacral and lumbar numbness	N/A	albuminocytological dissociation	MRI brain and whole spine with contrast: enhancement of the facial nerve	None	CR
8	Iftikhar et al.	Case report	1	1	None	36/M	No	Left	Moderna COVID-19 vaccine	2	1 day	facial weakness	left deltoid weakness, difficulty in speaking and eating, mild numbness and tingling of left arm, left subjective UL weakness	N/A	N/A	CT and MRI brain: unremarkable	oral Prednisolone and artificial tears	PR
9	Bonifacio et al.	LTE	5	1	N/A	66/M	Yes	B/L	Oxford-Astrazeneca	1	17 days	facial weakness, tongue and mouth numbness	paraesthesia of hands and feet	marked B/L LMN facial weakness. Tone, power and reflexes normal, but absent right ankle jerk. Light touch and pinprick sensation reduced symmetrically in B/L LL, gait ataxia	albuminocytological dissociation	MRI: unremarkable except for B/L smooth contrast enhancement along whole facial nerve	IVIg	PR
				2	N/A	43/M	Yes	B/L	same	1	17 days	severe facial weakness, dysphagia, dysguesia, tongue paraesthesia	myalgia, pins and needles in extremities, severe neck pain, urinary retention	severe B/L LMN facial weakness. Limb tone normal, mild weakness in right hip flexion. Reflexes were later lost. Flexor planter responses, Patchy, asymmetrical glove and stocking reduction in pinprick sensation, sensory ataxia.	cell count: elevated; Protein: elevated	MRI: unremarkable except for B/L smooth contrast enhancement along whole facial nerve	IVIg	PR
				3	N/A	51/M	Yes	Right, progessed to B/L	same	N/A	14 days	facial weakness	severe cramping leg pain, feet and hands numbness, spread to ankles	complete B/L LMN facial weakness. Tone, power and reflexes normal. Sensation impaired in all limbs, sensory ataxia.	albuminocytological dissociation	MRI: unremarkable except B/L smooth contrast enhancement along whole facial nerve	None	PR
				4	COVID-19 infection 5 weeks prior	71/F	Yes	B/L	same	N/A	15 days	facial weakness and dysguesia	lower back and abdominal pain, mild proximal leg weakness.	severe B/L LMN facial weakness, slight B/L weakness in hip flexion. Absent knee and left ankle reflexes, normal sensory	albuminocytological dissociation	MRI: unremarkable	None	PR
				5	N/A	53/M	Yes	B/L	same	N/A	14 days	facial and perioral paraesthesia progressing to severe simultaneous B/L facial weakness	lower back discomfort, radicular pain, LL parasthesia	severe LMN B/L facial weakness, normal power elsewhere. UL reflexes depressed; LL normal. Mild distal LL sensory loss to vibration and pinprick.	Albuminocytological dissociation	CT: unremarkable	None	PR
10	Nasuelli et al.	Case report	1	1	HTN and hyperuricemia	59/M	Yes	B/L	Oxford-Astrazeneca	1	10 days	facial diplegia, progressed to HB grade V	four limb distal paraesthesia, postural instability	physical exam: gait ataxia, global areflexia, distal UL and LL paraesthesia. No CN, vegetative, or sphincter involvement No spine sensory level.	albuminocytological dissociation	Brain and cervical MRI: unremarkable	IVIg and rehabilitation	PR
11	Burrows et al.	Case report	1	1	T2DM, HTN and hyperlipidemia	61/M	No	Right	Pfizer-BioNTech	1	5 h	right facial weakness	N/A	Right LMN facial palsy, lagophthalmos, no forehead movement	N/A	N/A	Prednisolone	CR
								Left		2	2 days	Severe left facial nerve palsy, dribbling and dysphagia	N/A	severe left facial nerve palsy HB grade IV, left lagophthalmos, SB grade 13, remainder of neurological exam and gait normal	N/A	N/A	Prednisolone	PR
12	Obermann et al.	Case report	1	1	N/A	21/F	No	Right	Pfizer-BioNTech	1	2 days	facial muscle paralysis	minimal muscle tenderness at injection site	N/A	unremarkable	MRI: unremarkable	oral Prednisolone, face muscle training, eye Protecting ointment and overnight eye patch.	PR
13	McKean et al.	Case report	1	1	dyslipidaemia	48/M	Yes	Left, progressed to B/L	Oxford-Astrazeneca	1	10 days	LMN facial weakness, initially HB grade III, progressed to grade V	severe mid-thoracic back pain	Progressive ascending paraesthesia, B/L LL weakness with foot drop, inability to bear weight, hand weakness and LL areflexia, impaired sensation to pain	cell count: elevated; Protein: elevated	MRI and CT brain: normal	IVIg, oral Prednisolone and physiotherapy	PR
14	Rossetti et al.	Case report	1	1	anxiety, depression, drinker and drug addict	38/M	Yes	B/L	J&J/Janssen COVID-19 vaccine	1	14 days	facial weakness, tongue and lips numbness and tingling, dysarthria, difficulty drinking from a straw and controlling his lips, cheeks, and tongue while eating	B/L hand and foot paresthesias	general exam: headaches, mild gait unsteadiness, generalized fatigue, exertional dyspneadysarthria, B/L LMN facial weakness, mild lagophthalmos B/L, inability to smile or puff cheeks against resistance. DTR reduced	albuminocytological dissociation	MRI brain: focal enhancement of the B/L internal auditory canal fundi and B/L cisternal segments of the trigeminal nerves	IVIg	PR
15	Čenščák et al.	Case report	1	1	bronchial asthma	42/M	Yes	Right	Pfizer-BioNTech	1	25 days	lagopthalmos	hands and feet paraesthesia, unsteady gait, weak knees, lumbalgia	right mimic muscle weakening and lagophthalmos up to 2 mm, handgrip and right arm elevation weak reflexes C5-8 minimal,L2-S2 absent, slow movement in lower limbs, hypoesthesia 10 cm above the wrist, B/L vibration sense mildly weak takes 3–4 steps by leaning, knees buckling and ataxia	albuminocytological dissociation	MRI LS spine with post-contrast: increased roots of cauda equina	IVIg and rehabilitation	PR
16	Prasad et al.	Case report	1	1	morbidly obese	41/M	Yes	B/L	J&J/Janssen COVID-19 vaccine	1	15 days	left facial droop, difficulty eating, right facial weakness	subjective weakness, distal paraesthesia, limb areflexia	CN: B/L LMN facial nerve palsy, more prominent on the left. B/L DTR absent at the patella and Achilles, mute plantar responses	albuminocytological dissociation	CT and MRI brain: colloid cyst MRI LS with contrast: thickening of cauda equina	IVIg and rehabilitation	PR
17	Christensen et al.	Case report	1	1	T2DM and Diabetic foot	73/M	Yes	B/L	Moderna COVID-19 vaccine	N/A	7 days	tingling in tip of tongue and around mouth, progressive B/L facial paresis and dysarthria	B/L sensory disturbances in LL, tingling in fingertips and dorsum of hands, left thoracic back pain radiating to the neck and jaw, could not walk	reflexes weakened, sensory ataxia in lower extremities.	unremarkable	MRI: unremarkable	None	PR
18	Rutkove et al.	Case report	1	1	None	58/M	Yes	B/L	J&J/Janssen COVID-19 vaccine	N/A	14 days	facial weakness	modest appendicular weakness, small subarachnoid hemorrhages	N/A	albuminocytological dissociation	N/A	N/A	N/A
19	Mason et al.	Case report	1	1	migraine headaches alcohol: 3 drinks per week	35/F	Yes	Right, progessed to B/L	Moderna COVID-19 vaccine	N/A	28 days	facial weakness and asymmetry, dysarthria, dysphagia, lagophthalmos	occipital headaches, mild right arm weakness	could not raise eyebrows, B/L lagophthalmos, unable to frown or smile. B/l N/Asolabial fold flattening, right handgrip weak CRanial nerves, touch, vibration, reflexes normal	albuminocytological dissociation	contrast MRI Brain and CT Angiography: unremarkable	IV methylprednisolone and acyclovir	PR
20	Corrêa et al.	Case report	1	1	None	42/M	Yes	Left	Oxford-Astrazeneca	1	7 days	left otalgia, facial muscles weakness, forehead muscles paralysis, lagophthalmos and labial hypomobility	None	N/A	unremarkable	Brain MRI with gadolinium: enhanced canalicular and labyrinthine portions of the left facial nerve and left geniculate ganglion	oral Prednisone	CR
21	Oo et al.	Case series	2	1	NSTEMI and seasonal influenza	51/M	Yes	B/L	Oxford-Astrazeneca	1	14 days	diplopia, dysphagia and bifacial weakness	lower back pain, Respiratory failure, LL motor deficit, progressive ascending LL sensorimotor deficit and areflexia	diplopia, bifacial weakness, moderate neck weakness, and flaccid areflexic quadriparesis with prominent proximal LL weakness. Pinprick sensation was distally reduced on the right LL	albuminocytologic dissociation	N/A	IVIg, MV and plasmapheresis	PR
				2	Renal Cell Carcinoma Atrial Fibrillation Hypercholesterolemia	66/M	Yes	Right	Oxford-Astrazeneca	1	21 days	NA	lower back pain, progressive ascending sensorimotor involvement -proximal LL weakness	Peoximal paraparesis, prominent right facial LMN weakness, and areflexia	Albuminocytologic dissociation	N/A	IVIg and rehabilitation	PR
22	Yu et al.	Case report	1	1	left Bell's palsy	36/F	No	Right	Sinovac	1	2 days	facial weakness and droop, eye discomfort, disappeared forehead wrinkles, lagophthalmos	None	CN: HB grade Ⅲ isolated right CN 7 palsy motor, sensory, and cerebellar examinations normal.	N/A	CT Brain: unremarkable	Prednisone, artificial tears, fluorometholone eye drops and acupuncture therapy	PR
23	Caro et al.	LTE	1	1	N/A	50/M	No	left	Pfizer-BioNTech	1	9 days	facial droop and effacement of left Nasolabial fold	tenderness at injection site.	complete left facial paralysis, lagopthalmos, unable to raise eye brow or left labial fissure.	N/A	MRI: intra cranial space occupying lesions and ischemic changes	Prednisone and articial tears.	
24	Ish et al.	LTE	1	1	None	50/M	No	Right	Covaxine	2	7 days	right lagopthalmos with redness and watering	None	Lagopthalmos with lower lid ectropion temporally. Drooping of angle of mouth, loss of nasolabial fold and wrinkiling of forehead.	N/A	N/A	topical antibiotics, lubricating eye drops and oral prednisone	
25	Karimi et al.	Case series	5	1	None	38/M	Yes	Right, progessed to B/L	Sputnik V	2	14 days	B/L facial numbness and weakness	numbness and parasthesias in distal UL	B/L Facial palsy HB grade IV	cell count: elevated; Protein: elevated	MRI: unremarkable	plasmapheresis	
				2	None	38/M	Yes	B/L	Sputnik V	1	6 days	B/L facial paralysis	LL weakness, decreased B/L sensation up to ankles and areflexia with flexor plantar responses.	B/L facial palsy HB grade IV and mild decreased LL light touch sensation and proprioception, distal to the ankle joints. Absent DTR	cell count: elevated; Protein: elevated	MRI brain and CS: few nonspecific B/L T2-hyperintensities viewed in the white matter	plasmapheresis	
				3	HTN	48/F	Yes	B/L	Sputnik V	1	17 days	B/L facial paralysis	generalized weakness with bulbar symptoms, dyspnea, and autonomic disorder.	Absent DTR.	N/A	N/A	Plasmapheresis and IVIg	
				4	HTN	44/M	Yes	B/L	Oxford-Astrazeneca	1	14 days	B/L facial numbness	LL parasthesia and weakness	flaccid tetraparesis	cell count: elevated	N/A	IVIg	
				5	HTN/CABG	79/M	Yes	B/L	Oxford-Astrazeneca	1	7 days	B/L facial paralysis	UL and LL parasthesia and weakness	B/L Facial palsy HB grade IV with UL and LL weakness and DTR absent.	N/A	N/A	Plasmapheresis	
26	Kanabar et al.	Case series	2	1	MS	61/F	Yes	B/L left > right.	Oxford-Astrazeneca	1	10 days	B/L LMN facial weakness (HB grade IV on the left and grade III on the right)	LL weakness, decreased B/L sensation up to ankles	N/A	N/A	N/A	IVIg	
				2	N/A	56/M	Yes	B/L	Oxford-Astrazeneca	1	7 days	B/L facial paralysis	severe back and LL radicular pain, LL numbness	B/L LMN facial weakness (HB grade IV on the left and grade III on the right) and decreased vibration sensation at the ankles. Areflexic plantar response.	N/A	N/A	IVIg	
27	Cellina et al.	Case report	1	1	N/A	35/F	No	left	Moderna COVID-19 vaccine	1	12 h	facial droop, dysphagia and lagophthalmos	None	slight asymmetry of the left corner of the mouth,	N/A	MRI:enhancement of left facial nerve.	oral Prednisone	
28	Walter et al.	LTE	1	1	None	30/F	No	left	Pfizer-BioNTech	2	2 months	left facial paralysis	ataxia	left facial paralysis, discreet right CN XII paralysis and massive ataxia of all extremities	Albuminocytological dissociation	MRI brain: weak flair hyperintensity of the brainstem, mesencephalon and cerebellar around the fourth ventricle without contrast. MRI of cervical and thoracic spine: unremarkable	IV cortisone with methylprednisolone	
29	Li Dang et al.	case report	1	1	N/A	63/M	Yes	B/L	Oxford-Astrazeneca	1	14 days	severe B/L facial weakness	sensory ataxia, facial diplegia involving forehead, proximal LL weakness	profound sensory ataxia, facial diplegia involving the forehead, proximal LL weakness, B/L LL areflexia and impaired distal LL proprioception, inability to walk without assistance and B/L lagophtalmos	albuminocytoloigcal dissociation	MRI contrast: B/L enhancement of CN III and CN VII, consistent with facial diplegia and partial B/L CN III palsy	IVIg	
30	Kharoubi et al.	Case report	1	1	Smoker	42/M	No	Right	Recommbinant vaccine	1	2 days	right facial assymtery	None	peripheral facial paralysis (Charles-Bell positive) HB grade III	N/A	N/A	Prednisone with eye care	
31	Badoiu et al.	LTE	1	1	N/A	53/F	Yes	B/L	Oxford-Astrazeneca	1	13 days	Asymmetric facial diplegia	tetrameric distal paraesthesia, progressive limb weakness.	mini mental motor deficit, hypopalasthesia, areflexia all 4 limbs, minimal ataxic gait. B/L latent blink reflex	albumino-cytological dissociation	MRI: unremarkable.	plasmapheresis	
32	Kulsirichawaroj et al.	Case report	1	1	None	16/F	No	Right, progessed to B/L	Pfizer-BioNTech	1	3 h	right facial numbness and drooling, right lagophthalmous, aguesia and difficulty furrowing right eyebrow	None	right facial hypoesthesia, complete right facial palsy, reduced taste in anterior 2/3rd of mouth and absent right gag reflex	N/A	MRI brain and CN: abnormal enhancement of right CN VII	IVIg	
33	Kim et al.	Case report	1	1	N/A	48/F	No	Left	Oxford-Astrazeneca	1	14 days	left facial paresis	band like pain in chest.	left facial paresis	N/A	N/A	IVIg and Prednisolone	
34	Mirmosayyeb et al.	Case series	2	1	N/A	27/F	No	Left	Sputnik V	1	5 days	left lagophthalmous, rightward deviation of left upper lip and unable to drink with straw	Pain at injection site and malaise.	facial nerve hemiparalysis	N/A	N/A	Prednisolone with valcyclovir	
				2	N/A	58/M	No	Left	Sputnik V	1	10 days	sudden left facial weakness and lagophthalmos, left mouth droop, drooling, aguesia, slurring of speech, tearing, inability to chew, smile, and move the left eyebrow	hyperthermia, myalgia, and pain in the injection site.	left facial paresis and lagopthalmos	N/A	N/A	Prednisolone and valacyclovir	
35	Mussatto et al.	case report	1	1	HIV and CKD	60/M	No	Left	Pfizer-BioNTech	1	42hrs	left facial droop and left lagophthalmos	NA	left facial weakness with forehead involved, inability to raise left eyebrow, sensation and strength intact in B/L UL and LL, mild exposure keratopathy, 5 mm lagophthalmos	N/A	N/A	oral Prednisone and valacyclovir	
36	Andreozzi et al.	Case series	2	1	Hashimoto thyroiditis	59/M	No	B/L	Oxford-Astrazeneca	1	15 days	B/L facial numbness	acute spontaneous burning pain of lower back, LL paraesthesia	complete facial diplegia and lagophtalmos: B/L loss of frontal forehead creases, could not raise eyebrows, and could not whistle or smile, mild dysarthria.	albuminocytologic dissociation	CT: unremarkable	IVIg	
				2	Atrial fibrillation	43/M	Yes	B/L	Oxford-Astrazeneca	1	7days	subacute facial pain and numbness with lagophthalmos	None	left prevalent facial diplegia; symmetrical weakness MRC grade 4+ in distal UL and LL muscles	albuminocytologic dissociation	N/A	IVIg	
37	Loza et al.	Case report	1	1	migraine	60/F	Yes	B/L	J&J/Janssen COVID-19 vaccine	1	18 days	B/L facial weakness and numbness	back and leg pain, headache, nausea, vomiting, and diplopia	Left eye esotropia, B/L ocular abduction deficit, areflexia and LL weakness	cell count: elevated; Protein: elevated	MRI LS:enhancement of cauda equina; Brain MRI:normal	IVIg	

Abbreviations: M, Male; F, Female; GBS, Guillian-Barre Syndrome; LTE, Letter to the Editor; CSF, Cerebrospinal fluid; N/A, Not Applicable; PR, Partial Recovery; CR, Complete Recovery; B/L, Bilateral; HTN, Hypertension, T2DM, Type 2 Insulin Independent Diabetes Mellitus; MRI, Magnetic Resonance Imaging; CT, Computed Tomography; LS, Lumbar Spine; CS, Cervical Spine; IVIg, Intravenous Immunoglobulin; IV, Intravenous; MV, Mechanical Ventilation; HB, House-Brackmann grading system; SB, Sunnybrook Facial Grading System; UL, Upper Limb; LL, Lower Limb; CN, Cranial Nerve; CN III, Occulomotor Nerve; CN VII, Facial Nerve, CN XII, Hypoglossal Nerve; LMN, Lower Motor Neuron; MS, Multiple Sclerosis; NSTEMI, Non ST Elevation Myocardial Infarction; CABG, Coronary Artery Bypass Graft; HIV, Human Immunodeficiency Virus; CKD, Chronic Kidney Disease; DTR, Deep Tendon Reflexes; J&J, Johnson & Johnson; MRC, Medical Research Council.

## 3 Results

Out of the 317 articles obtained through our database search, 231 were retrieved from Google Scholar while PubMed provided 86 results. 3 studies were extracted from other sources. After resolving any disagreements regarding study selection, studies were entered in an Excel sheet where 174 duplicate articles were removed. Subsequently, 146 articles were screened. 85 articles were rejected after perusing their titles and abstracts. In the final phase of selection, full-text versions of 61 studies were read for clarity. Articles were turned down for reporting aggregate-level data (n = 12), incomplete data on clinical progression (n = 8) and for being meta-analysis or systematic reviews (n = 4). Adhering to our rigorous criteria, 37 studies were finally chosen for inclusion in our systematic review. The detailed study selection procedure is given in a PRISMA flow chart in Supplementary File 3.

### 3.1 Patient characteristics

Our systematic review collated data from 58 individuals, from 37 studies, inflicted with facial palsy following COVID-19 vaccination. This data was obtained from 25 case reports, 5 case series and 7 LTEs. A higher occurrence of facial palsy was observed amongst the male gender in comparison to females. The ratio of male (n = 36) to female patients (n = 22) was 18:11. The mean age was 49.93 (SD: 14.16) years, ranging from 20 years to 79 years. Studies recorded data on comorbidities for only 39 individuals. Amongst those, 11 respondents (28.21%) had no known comorbidities. Hypertension (n = 8), Dyslipidaemia (n = 4), cardiac issues (n = 4) and Type 2 Diabetes Mellitus (T2DM) (n = 3) were, however, common comorbidities amongst those reported. It is also noteworthy that 10.26% of patients (n = 4) had a history of Bell's palsy or Guillain-Barré syndrome (GBS).

### 3.2 Symptom presentation

Table 1 displays the results of 58 patients who presented with facial palsy following their inoculation against COVID-19. Over half (51.72%) of the patients complained of facial paralysis following the Oxford-AstraZeneca vaccination. 15.52% had facial complaints after a Pfizer dose, whilst Moderna, Sputnik V and J&J/Janssen COVID-19 vaccine each had 8.62% of patients reporting the same. One patient each reported facial symptoms after Sinovac (1.72%) and Covaxine (1.72%). Thirty-two studies, bearing data from 51 patients, mentioned the vaccine dose which led to facial palsy. In 45 of these patients (88.24%), the onset of symptoms was after 1st dose, whereas only 5 (9.80%) had similar symptoms after the 2nd dose. Interestingly, one patient (1.72%) had the emergence of facial palsy after both his first and second vaccine jab. A majority (53.45%) had bilateral facial complaints. 18.97% and 17.24% had isolated left-sided and right-sided faces affected, respectively. Four patients had initial left (6.90%) and 1 patient (1.72%) had initial right-sided facial palsy which progressed to bilateral involvement over a course of time. The time of onset of facial palsy symptoms varied greatly, ranging from 3 h to 2 months after vaccination. Facial symptoms also varied in intensity per individual. From slight dysfunction to complete facial paralysis, patients experienced a myriad of symptoms. Common facial presenting complaints included lagophthalmos, dysgeusia, dysarthria, facial droop, loss of facial wrinkling, facial numbness, paraesthesia, otalgia, and tearing of eyes. Other complaints consisted of body ache (mainly chest and back region), limb paraesthesia and numbness (mainly hands and feet), fever, fatigue, headache, nausea, and ataxia.

### 3.3 Investigation and diagnostic results

CSF, MRI and CT Scan Brain and/or Spine were frequent investigations conducted by physicians. CSF analysis results were documented for 36 patients (62.07%). Albuminocytological dissociation was confirmed in approximately 2/3rd of patients (63.89%). Studies did not report the results of CT and MRI for 22 patients (37.93%). Amongst the remaining 36, enhancement of CN 7 was detected in the MRI Brain of 8 patients (22.22%). GBS was diagnosed in 67.24% of patients.

### 3.4 Treatment plan and its outcome

Intravenous immunoglobin (IVIg) (n = 27), corticosteroids (prednisolone, prednisone and methylprednisone) (n = 20) and Plasmapheresis (n = 8), were the first line of treatments. Antivirals (valacyclovir and acyclovir) (n = 5), eye care (eye drops and artificial tears) (n = 7) and rehabilitation measures (facial and physical) (n = 3) were oftentimes part of the treatment plan to foster faster recuperation. The outcome of the aforementioned treatment plan was documented for 54 patients across 34 studies. 75.93% patients (n = 41) partially recovered (PR)in the duration of the follow-up while 22.22% (n = 12) completely recovered (CR). 1 patient showed full improvement after his first onset of facial palsy following the 1st dose, but partial recovery after a recurrence of facial paralysis, after the 2nd dose. Partially recovered patients were either undergoing rehabilitation or continued the empiric treatment. It is also noteworthy that the follow-up period was insufficient, thus partially recovered patients should not be categorised as a failure in the treatment plan. Considering the rate of recovery in each patient, PR should instead be deemed as a favourable treatment outcome.

### 3.5 Pathophysiology

A temporal association between the COVID-19 vaccine and Bell's palsy has been accepted by numerous authors, but the pathogenesis is unclear. Genetic predisposition notwithstanding, viral infections, especially of the upper respiratory tract, are classically associated with demyelinating polyneuropathies. Damage may occur directly (autoimmune) or indirectly, compromising the blood supply; the vasa nervum (ischemia) or by degeneration of the myelin sheath (inflammation) [52].

Vaccines containing the viral vector, imitate the infection to trigger an exaggerated autoimmune response [59]. Antibodies generated against the virus protein, cross-react with the peripheral nerve proteins, causing demyelination. According to multiple speculations, this host antibody-antigen reaction may occur due to molecular mimicry. Similar vaccine epitopes, present in the myelin and axons, may spread by inflammation or superantigens. Vaccines also show an adjuvant effect, enhancing antigen presentation. Additionally, bystander activation of dormant self-antigens stimulates autoreactive T cells. Thus, causing an increased cell-mediated response [28,59]. Ozonoff et al. presented a valid discussion between the FDA Vaccines, Related Biologic Products Advisory Committee and Pfizer on the vaccines' likelihood to activate the body's innate immunity by the combination of mRNA and lipids. Hence, the interferons produced, interrupt the peripheral tolerance, causing neuropathy [15].

For non mRNA vaccines like Oxford-AstraZeneca and Janssen vaccine, the chimpanzee adenovirus vector directly attacks the culprit; SARS-CoV-2 spike protein, prompting more T cells activation. A cross-reaction follows, destroying the peripheral nerve upon sufficient exposure to the neuronal tissue. Elevated cytokines (IL-1, IL-6) and tumour necrosis factor (TNF a) were found in the patients with Bell's Palsy in comparison to a control group. Hence, proving the incidence of an aggravated cell-mediated response [31]. Sputnik V, a recombinant vector-based vaccine that uses adenovirus 26 (Ad26) and adenovirus 5 (Ad5) for molecular hijacking and expression also causes a similar immune-mediated reaction [30]. However, with immunogenetics specific to the individual, the HLA haplotype profile must not be disregarded in precipitating autoimmune neurological disorders [26].

Despite the inconclusive hypotheses, numerous patients found quick relief from IVIg therapy. Consequently, this directs us towards underlying immune-mediated pathogenesis holding the greatest probability [54].

## 4 Discussion

We reviewed the complete clinical course of 58 patients with symptomatic facial palsy following the COVID-19 vaccination. Besides the chief clinical features, patients often presented with accompanying body aches, fatigue, paraesthesia, and ataxia. These were also noted as major adverse events post-COVID-19 vaccination, in a study by Dutta et al. [17]. Of the reviewed cases, the majority were inoculated by the Oxford-AstraZeneca, a non-mRNA chimpanzee adenovirus vector vaccine, followed by 15% with Pfizer, an mRNA vaccine. Furthermore, these two vaccines have been linked to 15,538 (Pfizer) and 2751 (Oxford-Astra Zeneca) neurological adverse events, including facial palsy [17]. During phase 3 trials, 4 volunteers, who received the Pfizer vaccine, developed facial palsy as compared to zero in the control group [14]. However, this numerical imbalance was not reported with Oxford-AstraZeneca. Regardless, the numerous cases in our study, involving Oxford-AstraZeneca, warrant further exploration of the safety and efficacy of this vaccine.

Over half, 53.45%, of our recorded patients had bilateral facial palsy. Bell's Palsy is usually unilateral with an idiopathic aetiology whereas, bilateral is exceedingly rare, and secondary to systemic diseases like GBS [42]. This association must be credited as 67.24% of our patients were primarily diagnosed with GBS. Post-vaccination GBS has been analysed by several authors. The SARS-CoV 2 spike protein, in the vaccine, increases its transmission by binding to sialic acid-containing glycoprotein and gangliosides present on the neuronal cells' surface. After adequate exposure to the nerve components, antiganglioside antibodies are generated, ensuing in an autoimmune reaction. Thus, demyelination occurs after inflammatory changes, presenting with the afore-mentioned polyradiculopathy [26,27]. This could include the Facial Nerve (CN VII) of both sides, defining bilateral Bell's Palsy. Furthermore, CSF analysis shows albuminocytologic disassociation which can distinctively identify the acute inflammatory phase. An elevated protein level (normal is 0.55 g/L) in two-thirds of the patients, echoes nerve roots' inflammation [60].

In our sampled data, most patients complained of facial palsy after the first dose of vaccination (88%). While there is insufficient literature to explain the tapering rates of facial palsy in consecutive doses, several patients completed their vaccination after recovery. According to the demographic distribution, facial palsy affects people of all ages, with a peak incidence in patients in their 40s, similar to the observed mean age in our review (49.93 ± 14.16 years) [2]. The prevalence of Bell's palsy after immunization in this age group can be attributed to the higher reactogenicity of the COVID-19 vaccine among individuals between 18 and 65 years of age [17]. The reduced reactogenicity in patients above 65 years of age can be rationalized by immunosenescence; a series of age-linked changes in the soluble molecules that direct the maintenance and function of the immune system, the lymphoid organs that coordinate the maintenance of lymphocytes; and the initiation of immune responses [61] Ten per cent of patients also reported a recurrence of facial palsy; theoretically, because vaccination triggers a speedy shift from a subclinical to a symptomatic condition [59]. Other minor factors like a Human Immunodeficiency Virus (HIV) infection can also increase the risk of facial palsy [56]. Similarly, a history of facial palsy during 1st pregnancy and existing VZV-IgG antibodies can also manifest a potential reoccurrence [36].

Based on prior studies, about 5% of current patients are at high-risk for sequelae, especially synkinesis, incomplete or abnormal regeneration of the damaged facial nerve [3]. The most threatening consequence is permanent paralysis. Additionally, the malformation of nerve openings into different glands and ducts causes functional impairment.

Nonetheless, recovery is generally spontaneous from this adverse event following immunisation (AEFI). Maximally, 9 months have been observed for complete recuperation given an early and compliant corticosteroids course [16]. Oral corticosteroids, primarily prednisone, and IVIg therapy, have shown greater success than surgical management [1]. Corticosteroids are anti-inflammatory drugs, so the provided relief supports the inflammatory mechanism highlighted above. A favourable response to IVIg and Plasmapheresis indicated the cell's autoimmunity at play. Additionally, for lagophthalmos, treatment included eye drops, artificial tears, and temporary eye patches, to protect the vulnerable eye. Interestingly, a cohort study reported 16 more patients with acute bell's palsy during the pandemic in 2020 than in 2019, with a history of current or recent symptomatic COVID-19 infection [62]. Moreover, Tamaki et al. found an increased risk of Bell's palsy by 6.8% in individuals with COVID-19 infection versus those who were COVID-19 vaccinated [63]. Principally, SARS-CoV-2 has proved that its neurotropism is just one of the many strikes on multiple organ systems. Meanwhile, various authors emphasise that Bell's palsy has a high frequency of positive outcomes. The majority of our reviewed patients had partially recovered with hopes of full remission. The lack of follow-up in the reports hindered in providing an accurate analysis of the prognosis as many were undergoing treatment or within the expected duration noted for a complete symptomatic recovery.

## 5 Limitations

There were some limitations in the scope of our study, as case reports and series only assess a small number of patients. A total of 58 patients was insufficient to reach an accurate conclusion. A greater pool of patients with standardized reporting of clinical courses is needed for a definitive correlation between facial palsy and the COVID-19 vaccine. Larger, more robust studies must be analysed to assess the neurological side effects of the vaccines, particularly Oxford-AstraZeneca and Pfizer, with proper follow-up of the treatment course. This would reduce the reporting bias in the results and give better insight into the severity and prognosis of the condition. Nevertheless, a relationship between COVID-19 vaccines and the risk of facial palsy cannot be discounted.

## 6 Conclusions

Our review summarised all reported cases of facial palsy secondary to COVID-19 vaccination since the beginning of the pandemic. Oxford-AstraZeneca, a non-mRNA vaccine, was observed to account for most of the cases of facial palsy. A majority of patients were diagnosed with GBS, a demyelinating polyneuropathy, commonly presenting with bilateral facial palsy. Thus, any adverse event following immunisation must be explored and the possibility of such life-threatening disorders cannot be overlooked in clinical practice. Linking the relevant signs and symptoms to a COVID-19 vaccination history can ensure a prompt diagnosis and early management of facial palsy. Fortunately, facial palsy was seen to be a temporary disorder with extremely low chances of incidence in a larger sample size that could accurately reflect a population. With most individuals achieving complete recovery after appropriate therapy, we recommend that patients complete their vaccination after the condition has been resolved.

## Ethics approval

No ethical approval was required for this study.

## Funding

No financial support was received for this study.

## Authors’ contributions

Study conception and design: MK, IA, HA, HG. Study conduct and acquisition of data: AK, IU, HAC, AS. Data analysis: MK, IA, HA. Data interpretation: HG, IU, HAC. Drafting of the manuscript: AS, MYE, AK, MK, IA. Critical revision of the manuscript: IU, AS, HAC, MYE, MK. Final approval of the version to be published: All authors. All authors agree to be accountable for all aspects of the work.

## Consent

No consent was required for this study.

## Availability of data

The data that support the findings of this study are available from the corresponding author, HAC, upon reasonable request.

## Registration of research studies

Name of the registry: PROSPERO.

Unique Identifying number or registration ID: CRD42022328860.

Hyperlink to your specific registration: https://www.crd.york.ac.uk/PROSPERO/display_record.php?RecordID=328860.

## Provenance and peer review

Not commissioned, externally peer-reviewed.

## Guarantor

I, Mohammad Yasir Essar, the corresponding author for this review accept my role as the Guarantor for this research.

## Declaration of competing interest

The authors declare that they have no conflicts of interest and no financial interests related to the material of this manuscript.
