# Supplementary material for: Development of facial palsy following COVID-19 vaccination: A systematic review
Source: Ann Med Surg (Lond). 2022 Sep 30;82:104758. doi: 10.1016/j.amsu.2022.104758 (PMC9530738; doi:10.1016/j.amsu.2022.104758)
Supplement: Multimedia component 3 [file mmc3.docx]

# Supplementary File 3

## PubMed search string

1) For COVID-19 vaccine

"covid-19 vaccines"[MeSH Terms] OR ("covid-19"[All Fields] AND "vaccines"[All Fields]) OR "covid-19 vaccines"[All Fields] OR ("covid19"[All Fields] AND "vaccines"[All Fields]) OR "covid19 vaccines"[All Fields] OR "sars cov 2 vaccines"[All Fields] OR "covid-19 vaccines"[MeSH Terms] OR ("covid-19"[All Fields] AND "vaccines"[All Fields]) OR "covid-19 vaccines"[All Fields] OR "coronavirus disease 2019 vaccines"[All Fields] OR "2019-ncov vaccine mrna-1273"[MeSH Terms] OR ("2019-ncov"[All Fields] AND "vaccine"[All Fields] AND "mrna-1273"[All Fields]) OR "2019-ncov vaccine mrna-1273"[All Fields] OR "2019 ncov vaccine mrna 1273"[All Fields] OR "chadox1 ncov-19"[MeSH Terms] OR ("chadox1"[All Fields] AND "ncov-19"[All Fields]) OR "chadox1 ncov-19"[All Fields] OR "chadox1 ncov 19"[All Fields] OR "bnt162 vaccine"[MeSH Terms] OR ("bnt162"[All Fields] AND "vaccine"[All Fields]) OR "bnt162 vaccine"[All Fields].

AND

2) Facial palsy

"facial paralysis"[MeSH Terms] OR ("facial"[All Fields] AND "paralysis"[All Fields]) OR "facial paralysis"[All Fields] OR ("facial"[All Fields] AND "paresis"[All Fields]) OR "facial paresis"[All Fields] OR "facial paralysis"[MeSH Terms] OR ("facial"[All Fields] AND "paralysis"[All Fields]) OR "facial paralysis"[All Fields] OR ("peripheral"[All Fields] AND "facial"[All Fields] AND "paralysis"[All Fields]) OR "peripheral facial paralysis"[All Fields] OR ("central"[All Fields] AND "facial"[All Fields] AND "paralysis"[All Fields]) OR "central facial paralysis"[All Fields] OR "bell palsy"[MeSH Terms] OR ("bell"[All Fields] AND "palsy"[All Fields]) OR "bell palsy"[All Fields] OR ("bell's"[All Fields] AND "palsy"[All Fields]) OR "bell's palsy"[All Fields] OR "bell palsy"[MeSH Terms] OR ("bell"[All Fields] AND "palsy"[All Fields]) OR "bell palsy"[All Fields] OR ("idiopathic"[All Fields] AND "facial"[All Fields] AND "paralysis"[All Fields]) OR "idiopathic facial paralysis"[All Fields]

AND

3) ONLY HUMANS

This search yielded 86 results

## Google scholar search string

(COVID-19 vaccine OR SARS2 vaccine OR Coronavirus vaccine) AND (Facial palsy OR Bell's palsy OR facial paralysis OR facial paresis)

This search yielded 231 results.

**Joanna Brigg’s Institute Critical Appraisal Checklist for Case Reports**

| Author’s name | Q1 | Q2 | Q3 | Q4 | Q5 | Q6 | Q7 | Q8 | Total |
| --- | --- | --- | --- | --- | --- | --- | --- | --- | --- |
| Colella et al. [32] | Yes | Yes | Yes | Yes | Yes | Yes | No | Yes | 7/8 |
| Finsterer et al. [33] | No | Yes | Yes | Yes | Yes | Yes | No | Yes | 6/8 |
| Repajic et al. [34] | Yes | Yes | Yes | Yes | Yes | No | No | Yes | 6/8 |
| Nishizawa et al. [35] | Yes | Yes | Yes | Yes | No | No | No | Yes | 5/8 |
| Martin-Villares et al. [36] | Yes | Yes | Yes | Yes | Yes | Yes | No | Yes | 7/8 |
| Iftikhar et al. [7] | Yes | Yes | Yes | Yes | Yes | Yes | No | Yes | 7/8 |
| Bonifacio et al. [37] | Yes | Yes | Yes | Yes | No | Yes | No | Yes | 6/8 |
| Nasuelli et al. [38] | Yes | Yes | Yes | Yes | Yes | Yes | No | Yes | 7/8 |
| Burrows et al. [6] | Yes | Yes | Yes | Yes | Yes | Yes | No | Yes | 7/8 |
| Obermann et al. [5] | Yes | No | Yes | Yes | Yes | Yes | No | Yes | 6/8 |
| McKean et al. [39] | Yes | Yes | Yes | Yes | Yes | Yes | Yes | Yes | 8/8 |
| Rosetti et al. [40] | Yes | Yes | Yes | Yes | Yes | Yes | Yes | Yes | 8/8 |
| Čenščák et al. [41] | Yes | Yes | Yes | Yes | Yes | Yes | No | No | 6/8 |
| Prasad et al. [42] | Yes | Yes | Yes | Yes | Yes | Yes | No | Yes | 7/8 |
| Christensen et al. [43] | Yes | Yes | Yes | Yes | No | No | No | Yes | 5/8 |
| Rutkove et al. [44] | Yes | Yes | Yes | Yes | No | No | No | Yes | 5/8 |
| Mason et al. [45] | Yes | Yes | Yes | Yes | Yes | Yes | No | Yes | 7/8 |
| Corrêa et al. [46] | Yes | Yes | Yes | Yes | Yes | Yes | No | Yes | 7/8 |
| Yu et al. [47] | Yes | Yes | Yes | Yes | Yes | Yes | No | Yes | 7/8 |
| Caro et al. [48] | yes | yes | yes | yes | yes | yes | No | Yes | 7/8 |
| Ish et al. [49] | yes | yes | yes | No | Yes | Yes | No | Yes | 6/8 |
| Cellina et al. [4] | Yes | Yes | Yes | Yes | Yes | Yes | No | Yes | 7/8 |
| Walter et al. [50] | Yes | Yes | Yes | Yes | Yes | Yes | No | Yes | 7/8 |
| Li Dang et al. [51] | Yes | Yes | Yes | Yes | Yes | Yes | No | Yes | 7/8 |
| Kharoubi et al. [52] | Yes | Yes | Yes | No | Yes | Yes | No | Yes | 6/8 |
| Badoiu et al. [53] | Yes | Yes | Yes | Yes | Yes | Yes | No | Yes | 7/8 |
| Kulsirichawaroj et al. [54] | Yes | Yes | Yes | Yes | Yes | Yes | No | Yes | 7/8 |
| Kim et al. [55] | Yes | Yes | Yes | Yes | No | No | No | Yes | 6/8 |
| Mussatto et al. [56] | Yes | Yes | Yes | No | No | Yes | No | Yes | 6/8 |
| Loza et al. [57] | Yes | Yes | Yes | Yes | Yes | Yes | No | Yes | 7/8 |

**Joanna Brigg’s Institute Critical Appraisal Checklist for Case Series**

| Author’s name | Q1 | Q2 | Q3 | Q4 | Q5 | Q6 | Q7 | Q8 | Q9 | Q10 | Total |
| --- | --- | --- | --- | --- | --- | --- | --- | --- | --- | --- | --- |
| Maramattom et al. [25] | Yes | Yes | Yes | Yes | Yes | Yes | Yes | Yes | No | NA | 8/10 |
| Allen et al. [26] | Yes | Yes | Yes | Yes | Yes | Yes | Yes | yes | yes | NA | 9/10 |
| Oo et al. [27] | Yes | Yes | Yes | Yes | Yes | Yes | Yes | Yes | Yes | NA | 9/10 |
| Karimi et al. [28] | Yes | Yes | Yes | Yes | Yes | Yes | Yes | Yes | Yes | Yes | 10/10 |
| Kanabar et al. [29] | No | Yes | Yes | Yes | Yes | Yes | Yes | Yes | Yes | Yes | 9/10 |
| Mirmosayyeb et al. [30] | Yes | Yes | Yes | No | Yes | Yes | Yes | No | Yes | No | 8/10 |
| Andreozzi et al. [31] | Yes | Yes | Yes | No | Yes | Yes | Yes | Yes | Yes | Yes | 9/10 |

**Figure: PRISMA Flow chart of included studies.**

Identification

Studies extracted from database search (N=317)

Studies extracted from other sources (N=3)

Records after removal of duplicate studies (N=146)

Screening

Full text articles assessed for eligibility (N=61)

Studies screened (N=146)

Articles included in the systematic review (N=37)

Selection

Eligibility

Full text articles rejected with reasons (N=24)

Studies reported aggregate-level data (N=12)

Studies were meta-analysis and reviews (N=4)

Studies reported insufficient data regarding medical manifestation (N=8)

Records removed after reading title and abstract (N=85)
